# Psychosocial safety climate in Japanese workplaces

**DOI:** 10.1002/1348-9585.12430

**Published:** 2023-10-12

**Authors:** Maureen Dollard, May Young Loh

**Affiliations:** ^1^ PSC Global Observatory University of South Australia

Work stress is a global burden affecting millions of workers worldwide and should be prevented. In Japan, even though much effort has been given to reducing workplace psychosocial risks, there remains a high level of the tragic phenomena “karojisatsu” (work‐related suicides) and “karoshi” (death from overwork). More than half of Japanese workers are troubled with extreme work stress.^1^ Exploration of antecedents to workplace psychosocial risks (social factors that cause stress) is urgently needed to find solutions about how to prevent work stress and create psychological healthy workplaces.^2^ Thus far, work stress interventions have mostly highlighted individual‐focused strategies such as building individual resilience and personal coping strategies, putting the burden of solutions on individuals, or have focused on job redesign. These approaches neglect influential social context and structural factors, evident in the psychosocial safety climate of the organization.

Psychosocial safety climate (PSC) is a facet of organizational climate and a leading indicator of psychosocial risks and is therefore referred to as the “cause of the causes”, and positive employee health and work outcomes.^3^ Improving PSC in the workplace is likely to reduce stressful work conditions and undesirable psychological and physical injuries, as well as improve performance and employees' motivation. Studies of PSC assert that organizations are mostly hierarchical with power and influence largely coming from the top management team. The priorities, goals, and vision of executives and shareholders set the tone for the kinds of jobs on offer, and what organizations expect from their managers and workers, and thereby shape employees' behaviors and attitudes, and in turn affect their well‐being. For example, managers may set high‐performance work targets creating work pressure and overworking by employees, and in turn work stress. Protecting worker health and well‐being (i.e., via PSC) is important for workplace productivity and decent work. Building a high PSC context is in line with the United Nation's sustainable development goals of decent work and the International Labour Organization's Declaration on the Fundamental Principles and Rights at work.

By definition, PSC refers to the shared perceptions of employees about the “organisational policies, procedures and practices in relation to employee psychological health and safety”.^3^ PSC captures what an organization prioritizes and values, and is evident through organizational actions and commitment to protecting workers' psychological health. In the occupational health literature, job stress theories, such as the Job Demands‐Resources (JD‐R) theory,^4^ Job Demands‐Control (JD‐C)^5^ theory, and Effort‐Reward Imbalance (ERI)^6^ theory, have emphasized the role of job design, e.g., job demands and job resources, in affecting employees' outcomes. The JD‐R theory^4^ for instance proposes that job demands are those job aspects that require an individuals' effort and energy while job resources help one to accomplish job tasks. Extending this proposition, PSC theory argues that the design of a job is largely driven by the top management team and the organizational safety system that they design and promote. How PSC relates to employees' health and motivation can be explained through theoretical extensions to JD‐R theory, the extended, (a) health erosion pathway and (b) motivational pathway. The extended health erosion pathway of PSC theory proposes that at a high level of PSC, there will be a lower level of job demands and hence fewer employee psychological health problems such as depression and burnout. The extended motivational pathway proposes that at high PSC workers will be supplied with higher levels of resources which will motivate and inspire workers to engage more with their jobs. Since PSC is antecedent to psychosocial risks it is also clearly a predictor of the theoretical paths in JD‐C and ERI theories too, as well as psychosocial risks not included in those models, such as workplace bullying and harassment.

Efforts to study PSC in different cultures and nations are expanding. Although the concept of PSC was first introduced in Australia, literature has started to emerge in Asian countries, particularly Malaysia, but less so in countries from a Confucius background such as Japan, China, and Korea. In the first empirical study of PSC in Japanese workplaces, Inoue and colleagues^7^ examined the relationship between PSC and work engagement and psychological distress. Their study shines a spotlight on an initiative to shift the focus from traditional “psychological health as individual problems”, and job design issues, to organizational responsibility through PSC. Their occupational study involved a total of 2200 employees from different industries and occupations. Their findings clearly demonstrate that a high level of PSC relates to a lower level of psychological distress and a higher level of work engagement. Improving PSC in organizations therefore should be a target of work‐stress interventions.

An extremely important finding of their investigation is that PSC levels in Japanese workplaces are comparatively lower than in other countries. Based on PSC benchmarks,^8^ Japanese employees are working on average in an environment at high risk for future job strain and psychological health problems with PSC = 34.8 (range 12–60). PSC levels are lower than averages reported in Australia, Malaysia, New Zealand, and even the United States (US) which has few if any national regulations for worker psychological health.^9^ This finding accords with concerns raised about “karojisatsu” and “karoshi” in research and the media.

From a social perspective, this research highlights the parlous state of the Japanese work environment from the perspective of Japanese workers. Yet, across 45 different countries Japan ranks 6th in terms of expert ratings of national policy approaches to managing psychosocial factors at work (above Australia, Malaysia, New Zealand, and the US).^10^ Moreover, union density rates (i.e., the proportion of number of union workers versus total numbers of workers) also important for bargaining for better work conditions and PSC^11^ is as high for Japan (17%) as it is for New Zealand (18%) and higher than Australia (14%) and the US (10%).^12^ But these rates are not high (e.g., highest is Iceland at 92%). A disconnect between national policy approaches, such as low enforcement of regulations, low translation in the field, lack of capability in the field, and low union density could all underscore the low PSC ratings. It is also important to consider the role of national culture on social expectations, values, and norms at work (e.g., the role of individualistic vs. collectivistic values; power distance; uncertainty avoidance).

From a scientific perspective though, to strengthen the social impact of this research it is important to rule out competing explanations. Methodologically, response styles and propensity to respond positively or negatively across cultures could be an explanation for low PSC scores. For example, prior research found that Japanese employees have a tendency to suppress positive affect which might have led to lower self‐reported work engagement than in other countries^13^ and this issue could apply to PSC. Evidence that takes account of the potential influence of response styles and bias is therefore needed to uncover the reason for low PSC scores across the Japanese workforce.

While Inoue's paper enlightens us with evidence on Japanese workplaces, future research should consider replicating this study using a multilevel and multi‐wave design with data from various companies or organizations. The PSC construct concerns shared perceptions and consensus among the employees about their organizational climate. True to its theoretical conceptualization, PSC should be assessed as a group phenomenon, for example, by aggregating PSC individual perceptions to the company or work‐group level. What is likely assessed in Inoue's paper is “psychological” PSC (measured from the perspective of the individual) rather than “organizational” PSC (from the perspective of the organization). Since organizational PSC is derived from (aggregated) individual perceptions, it is likely that they are related but the results could be biased due to individual factors. Aggregating the data to the group level also helps to situate the source of the problem as belonging to the organization rather than individual because individual self‐report research is vulnerable to this attribution. That said there is theory and evidence that organizational and psychological PSC could exist simultaneously (the group has a collective experience, and the individual has an ideosyncratic experience) and both can be assessed in multilevel modeling.^14^ Repeated measures designs can also help tease out causal effects, providing evidence on the predictive value of PSC.

Scholars, practitioners, and organizations should also consider investing in interventions to build PSC.^15^ Introducing the principles of PSC to the management team, occupational health and safety representatives, and workers and their representatives (i.e., unions), and then enacting PSC principles should create an organization where humanity, decent work, and sustainability are highly valued. Such a workplace provides a stable platform for effective functioning, healthy work, and improved performance, enabling organizations to sustain and thrive in this ever‐changing working ecosystem. Inoue's paper leads us firmly in this positive direction.

## Data Availability

Data sharing is not applicable to this article as no new data were created or analyzed in this study.
